# The One‐Step Synthesis of Biologically Active Isochromans from Lignin‐Derived Aromatic Monomers in Tunable Deep Eutectic Solvents

**DOI:** 10.1002/chem.202502769

**Published:** 2025-12-05

**Authors:** Anjali Kottayi, Anastasiia M. Afanasenko, Antonio A. Castillo‐Garcia, Walid A. M. Elgaher, Jörg Haupenthal, Anna K. H. Hirsch, Katalin Barta

**Affiliations:** ^1^ Institute of Chemistry University of Graz Graz Austria; ^2^ Stratingh Institute for Chemistry University of Groningen Groningen The Netherlands; ^3^ Helmholtz Institute for Pharmaceutical Research Saarland (HIPS) − Helmholtz Centre for Infection Research (HZI) Saarbrücken Germany; ^4^ Department of Pharmacy Saarland University Saarbrücken Germany

**Keywords:** acidolysis, deep eutectic solvents, heterocycles, lignin, pharmaceuticals, sustainable chemistry

## Abstract

Clean synthetic strategies that embrace the inherent structural features of renewable building blocks to obtain biologically active compounds hold great potential to improve the economic viability of emerging biorefineries. Importantly, such an approach is highly beneficial for the sustainable manufacturing of pharmaceutically relevant molecules. Here, we demonstrate a one‐step protocol toward biologically active isochromans through the Oxa‐Pictet cyclization involving both aromatic aldehydes, including vanillin, and aliphatic aldehydes in combination with lignin‐derivable homovanillyl alcohol (**1**), a prominent aromatic platform chemical obtainable via diol‐assisted fractionation. Employing tunable deep eutectic solvents as benign reaction media allows for modulating reactivity under mild reaction conditions, and opens access to a library of isochromans in a single step. Several compounds exhibit promising properties, including PPI inhibition, anticancer, and antibacterial activities, highlighting the benefits of this synthetic strategy and its potential for drug discovery.

## Graphical abstract

A straightforward one‐step protocol enables the synthesis of bio‐based and biologically active isochromans through the Oxa‐Pictet cyclization involving diverse aldehydes, including vanillin in combination with lignin‐derivable homovanillyl alcohol, a prominent aromatic platform chemical obtainable via diol‐assisted fractionation of lignocellulose, employing tunable deep eutectic solvents as benign reaction media.

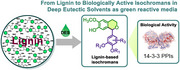

## 1 Introduction

Six membered *O*‐heterocycles are prominent scaffolds in diverse natural products and commonly found in pharmaceutically relevant molecules [1, 2, 3], thus, the atom‐efficient production of these moieties, preferably from renewable platform chemicals [4, 5] and using clean synthetic methods, would be highly advantageous.

**FIGURE 1:**
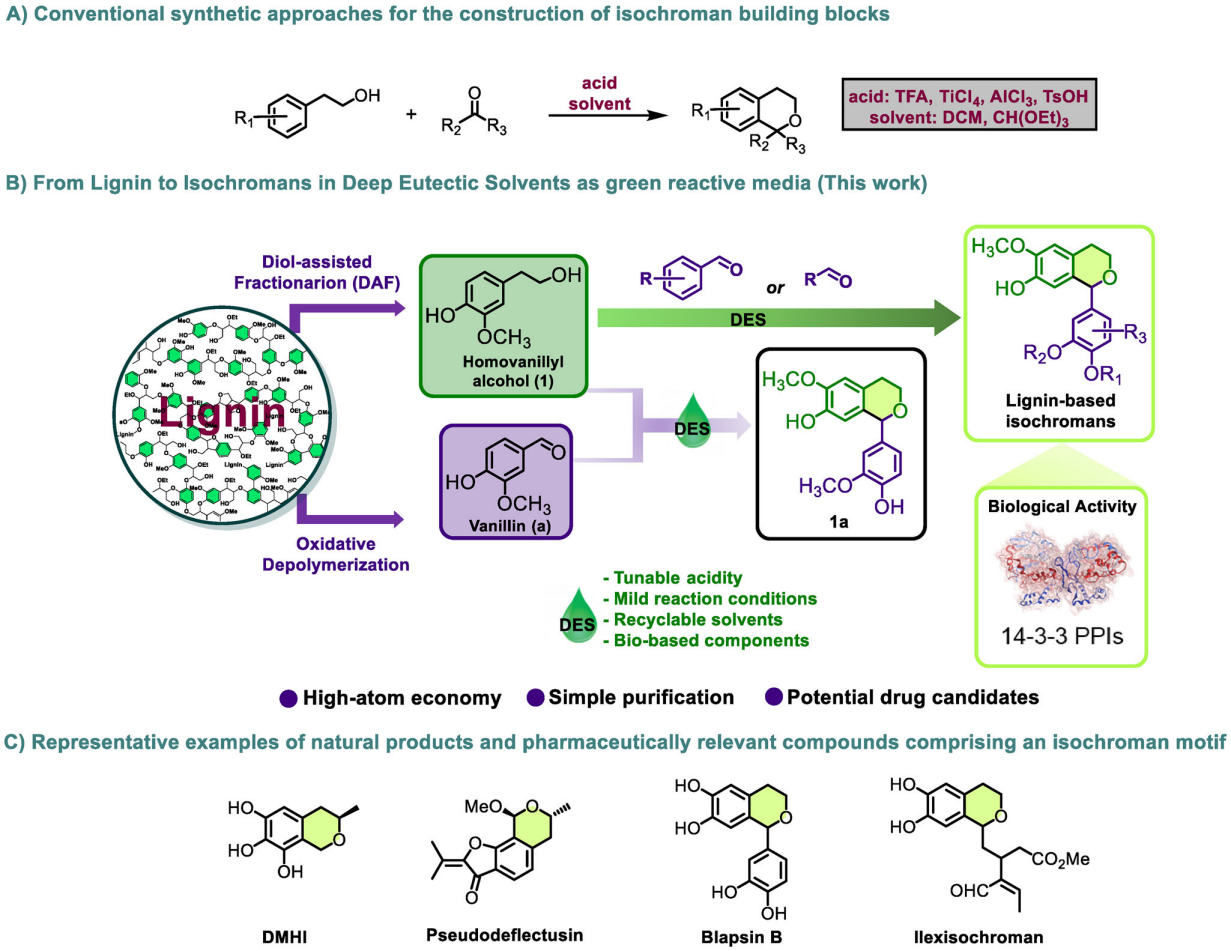
Sustainable synthetic methodology to obtain isochromans. (A) Conventional methods for the synthesis of isochromans using corrosive acids or toxic organic solvents.(B) The overall strategy of the work: construction of lignin‐derived isochromans from homovanillyl alcohol and vanillin via *oxa*‐Pictet–Spengler cyclization in deep eutectic solvent (DES). (C) Natural products and pharmaceutically relevant compounds containing an isochroman scaffold.

In this work we have devised novel strategies to obtain pharmaceutically relevant O‐heterocycles from lignin. We envisioned that lignin, the largest natural source of renewable aromatic building blocks, serves as an ideal starting material for the synthesis of *O*‐heterocycles given its intrinsic aromatic and highly oxygenated nature. Specific depolymerization strategies, including those developed in our group, would provide appropriate aromatic platform chemicals with suitable reactive groups, that enable the development of atom‐economic synthesis strategies for the production of pharmaceutically relevant compounds [6, 7]. Homovanillyl alcohol (**1**) can be selectively obtained through the diol‐assisted fractionation (DAF), an emerging ‘*lignin‐first’* methodology, pioneered in our group [8, 9, 10, 11]. This method operates through lignin acidolysis in conjunction with diol‐stabilization of the reactive C2‐aldehyde intermediates, and results in C2‐acetals, which undergo acetal deprotection/catalytic hydrogenation to **1**. Earlier, our group has demonstrated the synthesis of tetrahydroisoquinolines, quinazolin‐4(3H)‐ones and 3‐arylindoles starting from **1** [12], however six‐membered isochromans [13, 14, 15, 16], involving this intrinsic lignin‐derived general phenethyl moiety, were not yet explored. On the other hand, vanillin (**a**) is typically produced by the Cu‐catalyzed oxidative depolymerization of lignin under alkaline conditions [17, 18, 19]. This method has been industrially implemented (e. g. by the Borregaard company) [20, 21], and more recently a series of highly selective (electro)‐catalytic oxidative approaches starting from Kraft [22], or organosolv lignans have been reported [23]. Commonly applied in the food and cosmetic industries, vanillin has recently found application in a plethora of high‐performing polymer materials [24, 25, 26], however, the synthesis of pharmaceuticals from this platform chemical was considerably less explored [27]. Thus, we envisioned that the formation of a fully bio‐based six‐membered isochroman (**1a**), could be achieved via an *oxa*‐Pictet–Spengler cyclization, simultaneously involving aldehydes, such as the above‐mentioned vanillin (**a**), and homovanillyl alcohol (**1**). Our research focused on the development of a robust, one‐step methodology, involving deep eutectic solvents (DES) as alternative reaction media [28, 29, 30], to avoid hazardous volatile solvents or lengthy purification procedures [31, 32, 33] (Figure 1a and Supporting Information Section 8). Intriguingly, the synthesis of diverse heterocyclic motifs mediated by Bronsted‐acidic DES's has been previously reported, including the construction of pyrroles [34, 35, 36, 37], oxazoles [35], carbolines [36], among others. Thus, herein we envisioned that the synthesis of Isochromans in finely adjusted DES compositions, would also be possible. Indeed, we have shown that the acidity of the reaction medium, allows to control reactivity in the key cyclization step. Moreover, this sustainable approach enables access to a range of lignin‐based isochromans (Figure 1b), applying homovanillyl alcohol (**1**) in combination with vanillin (**a**) and its derivatives (**b**–**i**), as well as a range of aliphatic aldehydes (**j**–**n**). The obtained compound library, underwent testing for antibacterial, antioxidant, and anticancer properties as well as examining their ability to inhibit 14‐3‐3 protein‐protein interactions (PPIs) given their resemblance to Blapsin B, DMHI, among other biologically active compounds (Figure 1c) [38, 39, 40, 41].

## 2 Results and Discussion

DES are an emerging class of alternative reaction media that exhibit numerous advantages over conventional volatile organic solvents, typically used in pharmaceutical manufacturing. Besides negligible vapor pressure, biodegradability, low toxicity, and recyclability, these solvents display exquisite modularity and catalytic activity, depending on their composition [42, 43, 44, 45]. Given that the rate of Oxa‐Pictet–Spengler cyclization is dependent on the acidity of the reaction medium [46, 47, 48, 49], DES could be used as a benign ‘designer’ solvent in this transformation. Thus, we set out to investigate a range of natural DES, containing the combination of choline chloride (ChCl) as hydrogen bond acceptor (HBA) with a range of organic acids as hydrogen bond donor (HBD) in the construction of isochromans.

**FIGURE 2:**
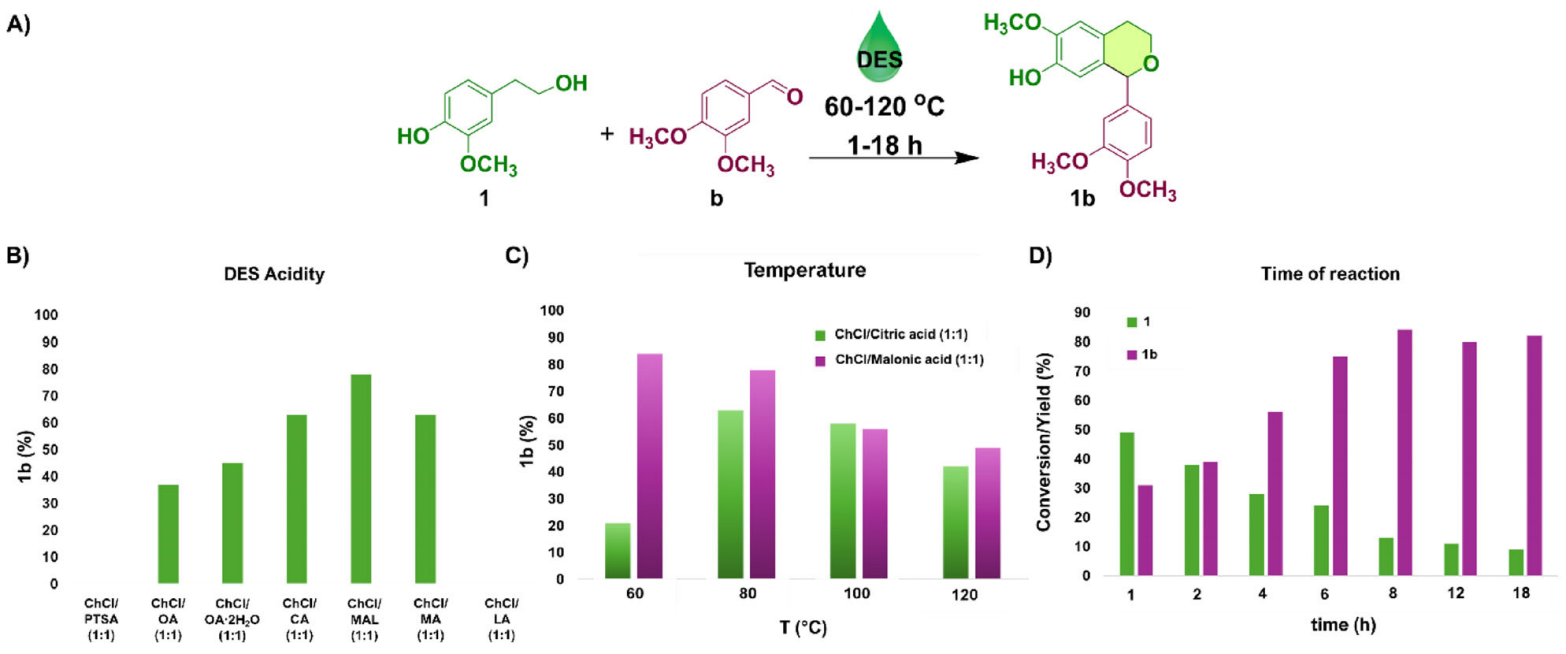
(A) Establishing the DES‐mediated construction of isochroman **1b** from Homovanillyl alcohol (**1**) and 3,4‐dimethoxyphenol (**b**). General reaction conditions: **1** (0.2 mmol), **b** (0.24 mmol, 1.2 equiv.), DESs (1:1 molar ratio, 1 g), 60–120 °C (heating block), 1–18 h. Yield and conversion were determined by ^1^H NMR with 1,2,4,5‐tetramethylbenzene as the internal standard. (B) Evaluation of different DES systems and pH dependence of the Oxa‐Pictet–Spengler cyclization. (C) Temperature dependence of DES pH (each pH value is depicted inside the graph bar). (D) Conversion and yield vs time of reaction. For numerical values see Supplementary Tables S1–3. Abreviations: PTSA (p‐toluene sulfonic acid), OA (oxalic acid), CA (citric acid), MAL (malonic acid), MA (malic acid), LA (lactic acid).

We started our investigation with DES compositions containing (ChCl), while systematically varying the nature of the organic acid components, and keeping the HBD:HBA ratio constant (1:1), for the model reaction of **1** with vanillin derivative 3,4‐dimethoxybenzaldehyde (**b**) (Figure 2a). As such, we successfully synthesized a range of DESs with diverse acidity, ranging from highly acidic **DES‐1** (ChCl: p‐TSA) to **DES‐7** (ChCl: Lactic acid) (Figure 2b). Satisfactorily, the efficiency of the reaction was found strongly dependent on the DES composition, showing an optimum performance when the Oxa‐Pictet–Spengler cyclization occurred in **DES 5** (ChCl: Malonic acid) enabling the synthesis of **1b** in a 78% yield (See Supporting Information, Section 2).

Neither highly acidic **DES‐1** nor the less acidic **DES‐7** showed the formation of the desired isochroman product **1b**. The best results were obtained with (**DES‐4**, 63%), (**DES‐5**, 78%), and (**DES‐6**, 63%), namely those compositions containing citric acid, malonic acid, and malic acid, respectively. Next, we studied the two best performing DES compositions, namely **DES‐4** (ChCl: Citric acid) and **DES‐5** (ChCl: Malonic acid) at reaction temperatures between 60 °C–120 °C (Figure 2c). Indeed, it is known from literature, that the physical properties of DESs, such as freezing point, viscosity, conductivity, are highly temperature‐dependent [42, 50, 51]. Furthermore, specific reports revealed a linear decrease of the pH with increasing temperature in DES [52]. Therefore, elevating the reaction temperature from 80 °C to 120 °C using **DES‐4** (ChCl: Citric acid), the acidity of the medium increased, leading to a drop in target product **1b** yield from 63% to 42%. A similar trend was observed using **DES‐5** (ChCl: Malonic acid), where the product yield dropped to 47% at 120 °C, showing also decomposition of malonic acid [53, 54].

Interestingly, a good conversion of 51% was observed already after 1 hour, albeit still displaying a modest product yield of 31%, whereas a high conversion and product yield were obtained already after 8 hours of reaction time, after which no significant change was observed (Figure 2d). Notably, the isolation of **1b** was also performed by a straightforward liquid‐liquid extraction with the addition of NaHSO_3_ for removal of unreacted aldehyde, affording a comparable yield to that using flash chromatography, representing a beneficial aspect in terms of process mass intensity (PMI), albeit the purity of 90% using this method was observed by NMR analysis (See Supporting Information Section 3) [55]. Furthermore, the recyclability of the DES has been also tested for the synthesis of **1b,** nevertheless, lower product yields were overall obtained when applying the recycled DES, due to the possible loss of malonic acid by decomposition. (See Supporting Information Section 4 for more details).

**FIGURE 3:**
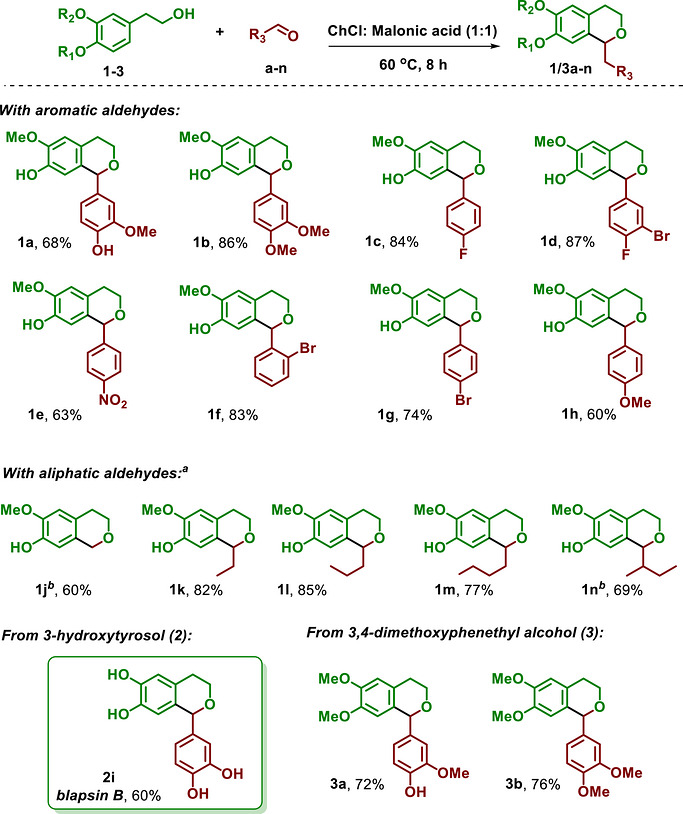
Construction of lignin‐derived isochromans using deep eutectic solvents. General reaction conditions: **1–3** (0.2 mmol), **a–n** (0.24 mmol, 1.2 equiv.), DESs (1:1 molar ratio, 1 g), 60 °C (heating block), 8 h. Isolated yields are shown.**^a^** reaction temperature = 80 °C.**^b^** time of reaction = 18 h.

With optimized reaction conditions at hand, a library of variously substituted isochromans were successfully synthesized. The coupling of **1** with benzaldehydes containing electron‐donating (**1a, 1b, 1 h**) and ‐withdrawing (**1c,1 g**) groups gave the desired *O*‐heterocycles in good to excellent isolated yields (Figure 3). To our delight, under an inert atmosphere, the reaction between 3‐hydroxytyrosol (**2**) and 3,4‐dihydroxybenzaldehyde (**i**) led to the formation of the natural product Blapsin B (**2i**) with a 60% isolated yield. Moreover, the synthesis of isochromans starting from homovanillyl alcohol derivative **3** was also explored, enabling the formation of **3a** and **3b** in good yields.

Finally, by employing aliphatic aldehydes as coupling partners, the target products **1j–n** were obtained in ≥ 60% yields and high purity after isolation.

### 2.1 Evaluation of Biological Activity of the Compound Libraries

Compounds with isochroman scaffolds exhibit various pharmacological effects such as antihypertensive, anti‐inflammatory agents, and are responsible for the modulation of central nervous system receptors, and display antimicrobial, antioxidant, and antitumor activities. Particularly, 14‐3‐3 is a class of regulatory proteins that interact with more than 1200 protein partners in mammalian cells, involved in cell proliferation and apoptosis. In human, seven 14‐3‐3 isoforms have been identified, namely beta (β), epsilon (ε), eta (η), gamma (γ), tau (τ), sigma (σ), and zeta (ζ). Modulation of 14‐3‐3 protein–protein interactions (PPI) represent a promising approach for treatment of cancer and neurodegenerative diseases [56]. Gratifyingly, isochroman‐based natural product blapsin, synthesized effectively using the described synthetic strategy, has been identified as a potent inhibitor of 14‐3‐3 γ [38]. It is also a naturally occurring constituent found in extra virgin olive oil as well as in the insect Blaps japanensis. Given the fact that our compounds share close structural similarity with blapsin B, we were interested in studying the binding properties of our isochroman derivatives to 14‐3‐3 protein. Accordingly, we first evaluated the binding interaction of the compounds with an immobilized 14‐3‐3 ζ using surface plasmon resonance (SPR) according to our previous work [56].

**FIGURE 4:**
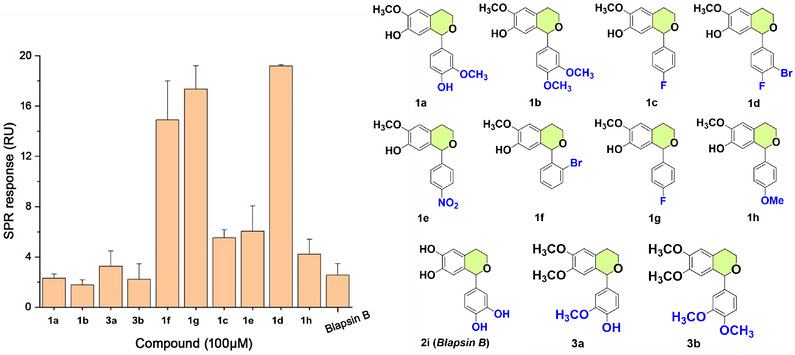
Normalized‐ SPR binding responses of the isochroman derivatives and blapsin B injected at concentration of 100 µM over an immobilized 14‐3‐3 ζ. Results obtained from two independent experiments.

Ten isochroman derivatives were injected at single concentration of 100 µM and their binding signals in resonance units (RU) were measured and compared to that of blapsin B at the same concentration. SPR binding response is directly proportional to the mass and number of protein‐bound molecules. To compensate for variations in molecular mass of the compounds, the RU values were normalized and results are shown in Figure 4. Isochroman derivatives displayed binding signals for 14‐3‐3 ζ equal to or higher than blapsin B with halogen‐containing derivatives **1f**, **1** **g**, **1d** showing the most promising binding affinity (See Supporting Information, Section 5). Such results indicate that lipophilic substituents such as bromine on the phenyl group at 1‐position of the isochroman ring improve the binding to 14‐3‐3, whereas hydrophilic groups such as hydroxyl, methoxy, and nitro are less favorable.

Quantitative and qualitative appraisal of synthesis of isochromans from fossil‐derived substrates and substrates derived from biomass based on the CHEM21 Metrics Toolkit at the first level: The case of **1a**. Finally, we decided to conduct the qualitative and quantitative appraisal of the green credentials for the synthesis of **1a** in terms of the CHEM21 Metrics toolkit at the first path level path [57] and the direct comparison with the method reported by Ussia and coworkers (See Table 1) [58]. The use of green solvents, dimethyl carbonate [59], and DES's [60], is highly convenient in both methods, however, the addition of H_2_SO_4_ is required in the case of dimethyl carbonate, whereas the ability to finely tune the acidity of the DES's mixture allows the formation of **1a** without using any additional reagent. Furthermore, the synthetic method reported by Ussia outperforms our approach in terms of energy requirement, enabling the synthesis of isochromans such as **1a** at room temperature, while the conditions for DES's mixtures (T = 60 °C) are higher, however, both approaches perform similarly during the work‐up step (liquid‐liquid extraction and flash chromatography). Additionally, both procedures display high atom economy (> 90%) albeit higher RME is observed for the DMC/H_2_SO_4_ system given the higher yield whereas a lower PMI is denoted for our approach due to the dual role of the DES's mixture as catalyst and reaction media (Table 2).

**TABLE 1:** Qualitative appraisal for the synthesis of **1a** in DMC/H_2_SO_4_ ref. [58] and DES's (This Work).

Approach	Solvent	Flag	Catalyst	Flag	Critical element	Flag	Energy	Flag	Work‐up	Flag	Health and Safety	Flag
Ref.^58^	DMC		H_2_SO_4_		—		rt		Liquid‐liquid extraction/ flash chromatography		—	
This work	ChCl/MA		—		—		60°C		Liquid‐liquid extraction/ flash chromatography		—	

*Note*: The acceptability of a particular process or reaction step is shown by a system of flags: green denotes ‘preferred’ whereas red is ‘undesirable’.

**TABLE 2:** Green metrics for the synthesis of **1a** in DMC/H_2_SO_4_ ref. 58 and DES's (This Work).

Approach	Yield (%)	AE (%)^a^	RME (%)^b^	OE (%)^c^	PMI (g g^−1^)^d^	PMI_RRC_ (g g^−1^)^d^
Ref. 58	80	94.3	68.8	72.9	34.6	1.5
This work	68	94.3	58.6	62.1	26.0	1.6

Abbreviations and equations:

^a^ AE (atom economy) = (molecular weight of product/total molecular weight of reactants) × 100.

^b^ RME (reaction mass efficiency) = (mass of isolated products/total mass of reactants) × 100.

^c^ OE (overall economy) = RME/AE × 100.

^d^ PMI (process mass intensity) = total mass process step (reagents + reactants + catalyst + solvent)/total mass product. PMI_RRC_ (process mass intensity) = total mass process step (reagents + reactants + catalyst)/total mass product.

Finally, a comparative study for the synthesis of homovanillyl alcohol starting from lignocellulosic biomass (a two‐step procedure) revealed the substantial decrease of the number of steps in comparison with potential synthetic procedures reported in the literature starting form benzene (8 steps procedure, See Supporting Information Section 6 and 7) [61, 62, 63, 64, 65, 66, 67, 68]. Moreover, the use of environmentally benign solvents such as DMC and H_2_O remarkably enhances the sustainability of the process in addition with the high selectivity observed in the lignin depolymerization step (Step 1) as well as the high yield obtained during the reduction step (Step 2). Furthermore, the purification of both acetal intermediate produced during step 1 and final homovanillyl alcohol is enabled via simple liquid‐liquid extraction. A similar case is observed for vanillin when comparing the biomass‐based process *versus* conventional methodologies starting from benzene, although the development of more efficient depolymerization strategies towards the more selective production of vanillin under milder conditions would be nonetheless advantageous [23]. Finally, as above mentioned, the synthesis of isochromans, including **1a**, has been previously reported under remarkably mild reaction conditions employing DMC as solvent and H_2_SO_4_ as catalyst at room temperature [57], however, the introduced DES‐mediated methodology could in principle outperform in terms of potential solvent recyclability.

## 3 Conclusion

Developing innovative, sustainable catalytic methods for producing pharmaceutical intermediates or biologically active molecules from nonedible renewable resources not only enhance the economic viability of lignocellulosic biorefineries, but also alleviates fossil dependence and tedious multistep synthetic procedures (See Supporting Information, Section 6,7) while aligning with environmental goals, fostering a greener and more sustainable future for pharmaceutical production. To the best of our knowledge, the application of Brønsted‐acidic DES for the synthesis of isochromans via the Oxa‐Pictet–Spengler cyclization has never been as yet reported. Based on this, our methodology represents a step forward toward the discovery of more sustainable alternatives for the synthesis of relevant heterocyclic compounds such as isochromans under mild and waste‐minimized conditions utilizing biomass resources such as lignin as platform chemicals, specifically homovanillyl alcohol and vanillin, whereby the scope was also extended to other alcohols and aldehydes. Our catalytic approach smartly incorporates the intrinsic functionalities of these monomers into the final structure of pharmaceutically relevant compounds.

By employing a benign reaction medium/catalyst under mild conditions, our methodology facilitates the synthesis of a diverse library of isochromans, including the natural product blapsin B. Finally, the preliminary evaluation of the biological activity of the novel compounds as 14‐3‐3 inhibitors has been also tested, encountering a promising activity for specific derivatives, however, further development of more active structures will be the object of study in the future. This represents a significant advancement in the field, offering both practical and environmental benefits, therefore, the development of these novel scaffolds opens up new opportunities for pharmaceutical research and development, underscoring the potential of using lignin‐derived monomers in green chemistry applications.

## 4 Experimental Section

### 4.1 Materials and Chemicals

Chemicals were obtained from Acros, Sigma–Aldrich or TCI at the highest available purity unless stated otherwise. When required, reactions were performed under nitrogen atmosphere using standard Schlenk techniques. DES were prepared by the reported procedures [45].

General procedure for the synthesis of isochromans from homovanillyl alcohol (**1**) and derivatives (2‐3) and aldehydes (a–n) in DES. An oven‐dried 20 mL vial equipped with a stirring bar was charged with alcohol 1–3 (0.2 mmol, 1 equiv.), aldehydes a–n (0.24 mmol, 1.2 equiv.) and ChCl: Malonic acid (1:1 molar ratio, 1 g), under air. Then the vial was capped, and the mixture was rapidly stirred at room temperature for 1 min, then it was heated to 60 °C and stirred for 8 h. After the reaction, the mixture was cooled down to room temperature and the pH was adjusted to 7.5 using saturated solution of NaHCO_3_.

The crude mixture was extracted with ethyl acetate (3×10 mL), passed through Na_2_SO_4_ and the solvent was removed in vacuo. The residue was purified by flash column chromatography using typically ethyl acetate and hexane (20:80) as eluent.

## Conflicts of Interest

The authors declare no conflict of interest.

## Supporting information




**Supporting file 1**: chem70507‐sup‐0001‐SuppMat.docx

## Data Availability

The data that support the findings of this study are available in the supplementary material of this article
