# Supplementary material for: The One‐Step Synthesis of Biologically Active Isochromans from Lignin‐Derived Aromatic Monomers in Tunable Deep Eutectic Solvents
Source: Chemistry. 2025 Dec 5;32(30):e02769. doi: 10.1002/chem.202502769 (PMC13472341; doi:10.1002/chem.202502769)
Supplement: Supplementary file 1 [file CHEM-32-e02769-s001.docx]

**Supplementary Information**

**The one-step Synthesis of Biologically Active Isochromans from Lignin-derived Aromatic Monomers in Tunable Deep Eutectic Solvents**

Anjali Kottayi,^1^ Anastasiia M. Afanasenko,^2^ Antonio A. Castillo-Garcia,^1^ Walid A. M. Elgaher,^3^ Jörg Haupenthal,^3^ Anna K.H. Hirsch^3,4^ and Katalin Barta^1*^

^1^Institute for Chemistry, University of Graz; Heinrichstrasse 28/II, 8010 Graz, Austria.

^2^ Stratingh Institute for Chemistry, University of Groningen, Nijenborgh 4, 9747 AG Groningen, The Netherlands

^3^Helmholtz Institute for Pharmaceutical Research Saarland (HIPS) − Helmholtz Centre for Infection Research (HZI); Campus Building E8.1, 66123 Saarbrücken, Germany.

^4^Saarland University, Department of Pharmacy, Campus Building E8.1, 66123 Saarbrücken (Germany)

*Correspondence to: katalin.barta@uni-graz.at

**1. General information**

Chemicals were obtained from Acros, Sigma-Aldrich or TCI at the highest available purity unless stated otherwise. When required, reactions were performed under nitrogen atmosphere using standard Schlenk techniques. Deep Eutectic Solvents (DES) were prepared by the reported procedures.^[1]^

**Column chromatography** was performed using Merck silica gel type 9385 230–400 mesh and typically dichloromethane and methanol or EtOAc and pentane as eluent.

**Thin layer chromatography (TLC):** Merck silica gel 60, 0.25 mm. The components were visualized by UV or KMnO_4_ staining.

**Gas Chromatography (GC)** was used for product identification as well as determination of conversion and selectivity values. Product identification was performed by GC-MS (5975C MSD) equipped with an HP-5 MS column, and helium as carrier gas. The temperature program started at 50 °C for 5 min, heated by 10 °C∙min-1 to 325 °C and held for 5 min. Conversion and products selectivity were determined by GC-FID (Agilent 8890 GC) equipped with an HP-5MS column using nitrogen as carrier gas.

**Nuclear Magnetic Resonance (NMR) spectroscopy:** ^1^H, and ^13^C NMR spectra were recorded on a Bruker Avance III 300 MHz (300 and 75 MHz, respectively) and 2D NMR spectra were recorded on a Bruker Avance III 700 MHz with Cryoplatform and a 5mm Triple-Resonance cryoprobe (700 and 175 MHz, respectively). ^1^H,^13^C NMR and 2D NMR spectra were recorded at RT. Chemical shift values are reported in ppm with the solvent resonance as the internal standard (CDCl_3_: 7.26 for ^1^H, 77.0 for ^13^C; CD_3_OD: 3.31 for ^1^H, 49.0 for ^13^C; DMSO-d_6_: 2.50 for ^1^H, 39.5 for ^13^C). Data are reported as follows: chemical shifts, multiplicity (s = singlet, d = doublet, t = triplet, q = quartet, br. = broad, m = multiplet), coupling constants (Hz), and integration.

**Standard techniques under inert atmosphere:** The synthesis of blapsin B was carried out under an argon atmosphere using oven (120 °C) dried glassware and standard Schlenk techniques as follows. To the prepared DES in a Schlenk tube, the solid materials were weighed under air and the Schlenk tube was subsequently connected to an argon line and vacuum-argon exchange was performed three times. The Schlenk tube was capped, and the mixture was rapidly stirred at room temperature for 1 min, then it was placed into a pre-heated metallic heating block to carry out the reaction at the indicated temperature for an indicated time.

**2. General procedures**

**2.1 General procedure for the synthesis of isochromans from homovanillyl alcohol (1) and aldehydes (a-n) in deep eutectic solvents (DESs)**

An oven-dried 20 mL vial equipped with a stirring bar was charged with homovanillyl alcohol (0.2 mmol, 1 equiv.), aldehyde (0.24 mmol, 1.2 equiv.) and ChCl: Malonic acid (1:1 molar ratio, 1 g), under air. Then the vial was capped, and the mixture was rapidly stirred at room temperature for 1 min, then it was heated to 60 °C and stirred for 8 h. After the reaction, the mixture was cooled down to room temperature and the pH was adjusted to 7.5 using saturated solution of NaHCO_3_. The crude mixture was extracted with ethyl acetate (3 x 10 mL), passed through Na_2_SO_4_ and the solvent was removed in vacuo. The residue was purified by flash column chromatography using typically ethyl acetate and hexane (20:80) as eluent.

**Table S1**: The influence of DESs acidity on the reaction yield.

| Entry | Abbreviation | DES | pH^a^ | Yield, [%]^b^ |
| --- | --- | --- | --- | --- |
| 1 | DES-1 | ChCl: *p*-TSA (1:1) | -1.85 | - |
| 2 | DES-2 | ChCl: Oxalic acid (1:1) | -1.51 | 37 |
| 3 | DES-3 | ChCl: Oxalic acid 2H_2_O (1:1) | -1.37 | 45 |
| 4 | DES-4 | ChCl: Citric acid (1:1) | -0.90 | 63 |
| 5 | DES-5 | ChCl: Malonic acid (1:1) | -0.70 | 78 |
| 6 | DES-6 | ChCl: Malic acid (1:1) | -0.33 | 63 |
| 7 | DES-7 | ChCl: Lactic acid (1:1) | 1.46 | - |

General reaction conditions: **1** (0.2 mmol), **b** (0.24 mmol), DESs (1:1 molar ratio, 1 g), 80 ^o^C (heating block), 8 h. ^a^pH measured using Mettler Toledo Seven compact digital pH meter. ^b^Yield was determined by ^1^H NMR with 1,2,4,5-tetramethyl benzene TMB as the internal standard.

**Table S2**: pH of DESs as a function of temperature and its effect on the yield of **3ab**.

| Entry | Abbreviation of DESs | pH | Temperature, [°C] | Yield, [%]^a^ |
| --- | --- | --- | --- | --- |
| 1 | DES-4 | -0.9 | 60 | 21 |
| 2 | DES-4 | -0.9 | 80 | 63 |
| 3 | DES-4 | -1.2 | 100 | 58 |
| 4 | DES-4 | -1.6 | 120 | 42 |
| 5 | **DES-5** | **-0.6** | **60** | **84** |
| 6 | **DES-5** | **-0.7** | **80** | **78** |
| 7 | DES-5 | 2.0 | 100 | 56 |
| 8 | DES-5 | 2.2 | 120 | 49 |

**General reaction conditions:** **1** (0.2 mmol), **b** (0.24 mmol, 1.2 equiv.) DESs (1:1 molar ratio, 1 g), 60-120 ^o^C (heating block), 8 h. ^a^Yield was determined by ^1^H NMR with 1,2,4,5-tetramethyl benzene TMB as the internal standard.

**Table S3**: Product formation profile as function of reaction time.

| **Entry** | **Time, [h]** | **Conversion, [%]** | **Yield, [%]** |
| --- | --- | --- | --- |
| 1 | 1 | 51 | 31 |
| 2 | 2 | 62 | 39 |
| 3 | 4 | 72 | 56 |
| 4 | 6 | 76 | 75 |
| 5 | 8 | 87 | 84 |
| 6 | 12 | 87 | 80 |
| 7 | 18 | 91 | 82 |

**General reaction conditions:** **1** (0.2 mmol), **b** (0.24 mmol, 1.2 equiv.) DES (ChCl: Malonic acid, 1:1 molar ratio, 1 g), 60 ^o^C (heating block), 1-18 h. ^a^Yield was determined by ^1^H NMR with 1,2,4,5-tetramethyl benzene (TMB) as the internal standard.

**2.2 General procedure for the synthesis of blapsin B**

An oven dried Schlenk tube was charged with 3-hydroxytyrosol (0.35 mmol, 1 equiv.), 3,4-dihydroxybenzaldehyde (0.42 mmol, 1.2 equiv.) and ChCl: malonic acid (1:1 molar ratio, 1 g), under air. The Schlenk tube was subsequently connected to an argon line and vacuum-argon exchange was performed three times. The Schlenk tube was capped and mixture was rapidly stirred at room temperature for 1 min, then it was heated to 60 °C and stirred for 8 h. After the reaction, the mixture was cooled down to room temperature and the pH was adjusted to 7.5 using saturated solution of NaHCO_3_. The crude mixture was extracted with ethyl acetate (3 x 10 mL), passed through Na_2_SO_4_ and the solvent was removed in vacuo. The residue was purified by flash column chromatography using typically ethyl acetate and hexane (20:80) as eluent.

**3. Liquid-liquid extraction protocol avoiding column chromatography**

An effective aldehyde and ketone removal protocol has been established that uses a simple liquid-liquid extraction step. In order to confirm this, the excess of aldehyde or ketone in the reaction mixture was converted into a charged bisulfite adduct using saturated solution of NaHSO_3_, and the final product was extracted into the organic phase using ethyl acetate.^[4]^

**General work-up procedure** (using the example of the synthesis of **1a** and **1b**): After completion of the reaction, the crude mixture was quenched with a saturated solution of NaHCO_3_ and extracted with ethyl acetate (3 x 10 mL). To remove excess of aldehyde used, the organic phase was further washed with a saturated solution of NaHSO_3_. The phases were separated; the organic phase was collected, and the solvent was removed on a rotary evaporator. Obtained crude residue was analysed by ^1^H NMR. According to the ^1^H NMR data, the aldehyde was removed almost completely, and the final product was obtained in more than 90% purity.

**Table S4**: Isolation of **1a** and **1b** by liquid-liquid extraction *versus* flash chromatography

| **1** | **a-b** | Isolated yield/ after liquid-liquid extraction  (%) | Purity (NMR) after liquid-liquid extraction  (%) | Isolated yield after flash chromatography  (%) |
| --- | --- | --- | --- | --- |
| **1**  0.2 mmol | **a**  (0.24 mmol) | 70 | 89 | 68 |
| **1**  0.2 mmol | **b**  (0.24 mmol) | 88 | 90 | 86 |

**Figure S1**: ^1^H NMR spectrum (CDCl_3_) of **1b** isolated using liquid-liquid extraction protocol.

**4. DES recyclability tests**

The synthesis of **1a** was performed three times using the same DES-mixture. After reaction completion and subsequent liquid-liquid extraction (water/ethyl acetate), the DES mixture was properly dried and the weight was measured before addition of new reagents observing a consistent mass of the DES media as well as isolated throughout three different reactions. Nevertheless, a lower amount of DES mixture was observed after reaction completion, which can be attributed to the decarboxylation of malonic acid during the drying step, consequently, lower yields were observed during cycle 2 and 3.

**Table S5**: Isolation of **1a** and **1b** by liquid-liquid extraction *versus* flash chromatography

|  | | | |
| --- | --- | --- | --- |
| Reaction Cycle | DES weight before reaction (mg) | DES weight after reaction completion/drying (mg) | Isolated yield (%)^[a]^ |
| 1 | 1000 | 989 | 68 |
| 2 | 989 | 940 | 56 |
| 3 | 940 | 921 | 47 |
| General Reaction Conditions: **1** (0.2 mmol), **a-b** (0.24 mmol), DES (1:1 ratio, 1000 mg), 60 C, 8 h.  ^[a]^ Isolated yield for **1a** is shown | | | |

**5. Surface plasmon resonance (SPR) binding studies**

The SPR experiments were performed using a Reichert 4SPR surface plasmon resonance spectrometer (Reichert Technologies, Buffalo, NY, USA), and medium density carboxymethyl dextran hydrogel CMD500M sensor chips (XanTec Bioanalytics, Düsseldorf, Germany). Doubly distilled (dd) water was used as the running buffer for immobilization. HBS-EP buffer (10 mm HEPES, 150 mm NaCl, 3 mm EDTA, 0.005% v/v tween 20, pH 7.4) containing 5% v/v DMSO was used as the running buffer for binding studies. All running buffers were filtered and degassed prior to use. The 14-3-3 ζ-isoform (30.3 kDa) was immobilized in one of the four flow channels via amine-coupling according to a reported protocol.^1^ Another flow channel was left blank to serve as a reference. The system was initially primed with borate buffer 100 mm (pH 9.0), then the carboxymethyldextran matrix was activated by a 1:1 mixture of *N*-ethyl-*N*′-(3-dimethylaminopropyl)carbodiimide hydrochloride (EDC) 100 mm and *N*-hydroxysuccinimide (NHS) 100 mm at a flow rate of 10 µL/min for 7 min. The 14-3-3 protein was diluted to a final concentration of 57 μg/mL in 10 mm sodium acetate buffer (pH 4.6) and was injected at a flow rate of 10 µL/min for 7 min. Non-reacted surface was quenched by 1 m ethanolamine hydrochloride (pH 8.5) at a flow rate of 10 µL/min for 8 min. A series of 10 buffer injections was run initially on both reference and active surfaces to equilibrate the system resulting in a stable immobilization level of approximately 2700 µ refractive index unit (μRIU). Binding experiments were performed at 20 °C. For binding study, blapsin B and the compounds dissolved in DMSO were diluted with HBS-EP buffer to a final concentration of 100 µM with 5% v/v DMSO and were injected at a flow rate of 40 μL/min. For determination of equilibrium dissociation constant (*K*_D_), single-cycle kinetics were applied using six concentrations of the compounds (400–12.5 µM) via two-fold serial dilutions. The association time was set to 60 s, and the dissociation phase was recorded for 120 s. Differences in the bulk refractive index due to DMSO were corrected by a calibration curve (five concentrations: 4–6% v/v DMSO in HBS-EP buffer). Data processing and analysis were performed by Scrubber software (Version 2.0c, 2008, BioLogic Software). Sensorgrams were calculated by sequential subtractions of the corresponding curves obtained from the reference flow channel and the running buffer (blank). SPR responses are expressed in resonance unit (RU). For normalization of responses at 100 µM, the RU values of the analytes were subsequently multiplied by 350 and divided by the corresponding molecular weights. The *K*_D_ values were calculated by global fitting of the association and dissociation curves. Data were obtained from two independent experiments.

**Table S6.** The kinetic parameters of compounds **3af**, **3ag**, and **3ad** binding to 14-3-3.*^a^*

| Compound | *R_max_* (RU) | *K*_a_ (m^-1^s^-1^) | *K*_d_ (s^-1^) | *K*_D_ (µm) |
| --- | --- | --- | --- | --- |
| **1f** | 139 ± 9 | (6.6 ± 0.7) × 10^2^ | 0.44 ± 0.03 | 660 ± 60 |
| **1g** | 145 ± 8 | (6.7 ± 0.6) × 10^2^ | 0.50 ± 0.03 | 740 ± 60 |
| **1d** | 171 ± 4 | (4.6 ± 0.2) × 10^2^ | 0.306 ± 0.006 | 660 ± 20 |

*^a^* *R_max_*: maximum analyte binding capacity; *k*_a_: association rate constant; *k*_d_: dissociation rate constant; *K*_D_: equilibrium dissociation constant.

| **A** | **B** |
| --- | --- |
|  |  |
| **C** | |
|  | |

**Figure S2**: Sensorgrams overlay of compounds **1f** (A), **1g** (B), and **1d** (C) injected at concentrations of 12.5–400 µM over an immobilized 14-3-3 ζ. Global fitting of the association and dissociation curves (red traces).

**6. Comparison of the synthesis of C2-isochromans from fossil fuels and lignocellulose**

**6.1** Synthesis of homovanillyl alcohol:

Literature-based approach: selection of pathways based on the available literature, Step 1,^[5]^ Step 2,^[6]^ Step 3,^[7]^ Step 4,^[8]^ Step 5,^[9]^ Step 6,^[10]^ Step 7,^[11]^ Step 8.^[11]^

**6.2** Synthesis of vanillin:

Literature-based approach: selection of pathways based on the available literature, Step 1,^[5]^ Step 2,^[6]^ Step 3,^[7]^ Step 4,^[8]^ Step 5,6, 7.^[12]^

**6.3** Conventional procedure for the *oxa*-Pictet-Spengler cyclization:^[13]^

**6.4** Synthesis of homovanillyl alcohol from biomass in two steps: ^[2,3]^

**6.5** Synthesis of vanillin from biomass via oxidative depolymerization:^[14]^

**6.6** Sustainable synthesis of isochromans from biomass-derived substrates

**7** **Quantitative and qualitative appraisals of synthesis of isochromans from fossil derived substrates and substrates derived from biomass based on the CHEM21 Metrics Toolkit at the first level: The case of 1a**

| **1. Synthesis of homovanillyl alcohol (1)** | | | | | | | | | | | | | | |
| --- | --- | --- | --- | --- | --- | --- | --- | --- | --- | --- | --- | --- | --- | --- |
| Step | Yield  (%) | | AE  (%) | | Solvent  Rxn | Flag | Critical  Element | Flag | Reagent  Use | Flag | Energy  Use | Flag | Work-  up | Flag |
| 1 | 91 | | 99 | | - |  | Al |  | catalyst |  | 150 °C |  | Distill. |  |
| 2 | 94 | | 99 | | - |  | - |  | catalyst |  | 118-126 °C |  | Distill. |  |
| 3 | 96 | | 62 | | HOAc |  | - |  | catalyst |  | 22 °C |  | Filtr. |  |
| 4 | 54 | | 86 | | H_2_O |  | S |  | catalyst |  | 50 °C |  | Distill. |  |
| 5 | 82 | | 42 | | Acteone |  | I |  | catalyst |  | 41 °C |  | Chrom. |  |
| 6 | 35 | | 81 | | H_2_O |  | Cu |  | catalyst |  | 25 °C |  | Distill. |  |
| 7 | 80 | | 78 | | MeOH:DCM  (1:1) |  | - |  | catalyst |  | -78 °C |  | Extr. |  |
| 8 | 80 | | 82 | | MeOH:DCM  (1:1) |  | B |  | Stoich. |  | RT |  | Extr. |  |
| **2. Synthesis of vanillin (a)** | | | | | | | | | | | | | | |
| Step | | Yield  (%) | | AE  (%) | Solvent  Rxn | Flag | Critical  Element | Flag | Reagent  Use | Flag | Energy  Use | Flag | Work-  up | Flag |
| 1 | 91 | | 99 | | - |  | Al |  | catalyst |  | 150 °C |  | Distill. |  |
| 2 | 94 | | 99 | | - |  | - |  | catalyst |  | 118-126 °C |  | Distill. |  |
| 3 | 96 | | 62 | | HOAc |  | - |  | catalyst |  | 22 °C |  | Filtr. |  |
| 4 | 54 | | 86 | | H_2_O |  | S |  | catalyst |  | 50 °C |  | Distill. |  |
| 5 | 86 | | 100 | | H_2_O |  | Al |  | catalyst |  | 60 °C |  | Quench./  Extr. |  |
| 6 | 44 | | 75 | | EtOAc |  | Cu |  | excess |  | 60 °C |  | Extr. |  |
| 7 | 94 | | 57 | | DCM |  | S |  | excess |  | 60 °C |  | Chrom. |  |
| **Literature based approach for the synthesis of isochromans from homovanillyl alcohol and vanillin** | | | | | | | | | | | | | | |
| Step | Yield  (%) | | AE  (%) | | Solvent  Rxn | Flag | Critical  Element | Flag | Reagent  Use | Flag | Energy  Use | Flag | Work-  up | Flag |
| 1 | 80 | | 94 | | DMC |  | S |  | catalyst |  | RT |  | Chrom. |  |

| **Our developed approach for obtaining homovanillyl alcohol from biomass** | | | | | | | | | | | | |
| --- | --- | --- | --- | --- | --- | --- | --- | --- | --- | --- | --- | --- |
| Step | Yield  (%) | AE  (%) | Solvent  Rxn | Flag | Critical  Element | Flag | Reagent  Use | Flag | Energy  Use | Flag | Work-  up | Flag |
| Biomass processing  (Step 1) | | | DMC |  | S |  | catalyst |  | 140 °C |  | Extr. |  |
| 2 | 92 | 79 | H_2_O |  | Ru |  | catalyst |  | 90 °C |  | Extr. |  |
| **Obtaining vanillin from biomass** | | | | | | | | | | | | |
| Biomass processing | | | H_2_O |  | - |  | - |  | 160 °C |  | Extr. |  |
| **Our developed approach for the synthesis of isochromans from homovanillyl alcohol and vanillin** | | | | | | | | | | | | |
| 2 | 68 | 94 | DES |  | - | - | - | - | 60 ° C |  | Extr. |  |

Abbreviations: Chrom.: chromatography; Distill.: distillation; Extr.: extraction; Filtr.: filtration; Quench.: quenching; Rxn.: reaction; Stoich.: stoichiometric.

**8. Summary of reported synthesis of isochromans using commercially available acids via *oxa*-Pictet-Spengler cyclization**

**Table S7**: Reagents and solvents used in the conventional synthesis of isochromans as reported in the literature.

| Entry | Reagents | Solvent | Reaction conditions | Yield (%) | Reference |
| --- | --- | --- | --- | --- | --- |
| 1 | Fe(OTf)_2_ | Toluene | 70 °C, 4 h | 98 | Zhou, Wang et al. (2018)^[15]^ |
| 2 | PTSA | Toluene | 110 °C, 18 h | 60-70 | Kapadia, Harding (2013)^[16]^ |
| 3 | TfOH | HFIP | rt, 1 h | 70 | Muller et al. (2023)^[17]^ |
| 4 | PTSA, Na_2_SO_4_ | MeOH | 38 °C, 48 h-7 days | 40-60 | Guiso, Betrow et al. (2008)^[18]^ |
| 5 | PTSA,  MS | MeOH | 4 °C, 24 h | 60-98 | Guiso, Bianco et al. (2003)^[19]^ |
| 6 | PTSA | MeOH | 64 °C, 3-6 h | - | Lorenz, Zeh et al. (2005)^[20]^ |
| 7 | Bi(OTf)_3_ | Toluene | 80-110 °C, 1-2 h | Up to 99 | Bouguerbe, Hoffman et al. (2010)^[21]^ |
| 8 | Bi(OTf)_3_ | DCE | 456 nm irradiation, 15 h | 86 | Tanaka, Mitton-Fry et al. (2024)^[22]^ |
| 9 | PTSA | H2O | 110 °C, 1 h | 55-98 | Saeed et al. (2010)^[23]^ |
| 10 | TMSOTf | Anhyd DCM | 0 °C-rt | 40-70 | Sarkar, Sultana et al. (2016)^[24]^ |
| 11 | PTSA, Mg(SO)_4_ | MeCN | 20 °C | 78 | Miles, Heinsohn et al. (2002)^[25]^ |
| 12 | BF_3_.OEt_2_ | DCM | 0-20 °C  inert conditions | 91 | Verma, Chennaiah et al. (2018)^[26]^ |
| 13 | Bi(OTf)_3_ | Toluene | 20-80 °C | 91 | Bouguerbe, Lherbet et al. (2010)^[27]^ |
| 14 | PFOS | HFIP in H_2_O | 90 °C, 18 h | 86 | Saito, Takayama et al. (2007)^[28]^ |
| 15 | PTSA | DCM | Rt, 9-12 h | 71-86 | Reddy, Burra et al. (2016)^[29]^ |
| 16 | H_2_SO_4_ in nano silica | EtOH | 80 °C, 0.5 h | 40-90 | Khorsandi, Khosropour et al. (2011)^[30]^ |
| 17 | H_2_SO_4_ | DMC | rt, 2-24 h | 60-98 | Bernini, Crisante et al. (2016)^[13]^ |

**9. Characterization data of isolated compounds**

**1-(4-hydroxy-3-methoxyphenyl)-6-methoxyisochroman-7-ol (1a)**

The compound was synthesized according to the **General procedure**. Homovanillyl alcohol (34 mg, 0.2 mmol) affords **1a** (41 mg, 68% yield). White semi-solid was obtained after column chromatography (SiO_2_, Hexane/EtOAc 70:30 to 40:60). **^1^H NMR** (300 MHz, CDCl_3_) δ 6.85 (d, *J* = 8.5 Hz, 1H), 6.79 (d, *J* = 7.5 Hz, 2H), 6.62 (s, 1H), 6.33 (s, 1H), 5.69 (s, 1H), 5.55 (s, 1H), 5.50 (s, 1H), 4.17 (ddd, *J* = 11.5, 5.6, 3.8 Hz, 1H), 3.92 (dd, *J* = 9.2, 5.2 Hz, 1H), 3.85 (d, *J* = 14.6 Hz, 6H), 3.06 (ddd, *J* = 15.5, 9.6, 5.7 Hz, 1H), 2.69 (dt, *J* = 16.0, 3.9, 3.9 Hz, 1H). **^13^C NMR** (75 MHz, CDCl_3_) δ 146.7, 145.6, 145.5, 143.7, 134.2, 130.3, 125.3, 122.2, 113.9, 112.7, 111.0, 110.5, 79.4, 64.1, 56.0, 56.0, 28.5. **HRMS** (APCI^+^, m/z) calculated for C_17_H_19_O_5_ [M+H]^+^: 303.1154; found: 303.1230.

**1-(3,4-dimethoxyphenyl)-6-methoxyisochroman-7-ol (1b)**

The compound was synthesized according to the **General procedure**. Homovanillyl alcohol (59 mg, 0.35 mmol) affords **1b** (95 mg, 86% yield). White semi-solid was obtained after column chromatography (SiO_2_, Hexane/EtOAc 80:20). **^1^H NMR** (300 MHz, CDCl_3_) δ 6.81 (d, *J* = 2.5 Hz, 3H), 6.61 (s, 1H), 6.32 (s, 1H), 5.54 (d, *J* = 10.7 Hz, 2H), 4.16 (ddd, *J* = 11.3, 5.5, 3.7 Hz, 1H), 3.92 (dd, *J* = 6.0, 3.2 Hz, 1H), 3.84 (d, *J* = 11.1 Hz, 9H), 3.05 (ddd, *J* = 15.4, 9.5, 5.6 Hz, 1H), 2.68 (dt, *J* = 16.0, 3.9, 3.9 Hz, 1H). **^13^C NMR** (75 MHz, CDCl_3_) δ 149.1, 148.9, 145.5, 143.7, 134.8, 130.2, 125.3, 121.4, 112.7, 111.6, 110.7, 110.5, 79.3, 64.1, 56.0, 55.9, 28.5. **HRMS** (APCI^+^, m/z) calculated for C_18_H_21_O_5_ [M+H]^+^:317.13107; found:317.138639.

**1-(3,4-dimethoxyphenyl)-6-methoxyisochroman-7-ol (3a)**

The compound was synthesized according to the **General procedure**. Homovanillyl alcohol (59 mg, 0.35 mmol) affords **2a** (79 mg, 72% yield). White semi-solid was obtained after column chromatography (SiO_2_, Hexane/EtOAc 80:20). **^1^H NMR** (300 MHz, CDCl_3_) δ 6.83 (d, *J* = 7.9 Hz, 1H), 6.76 (dd, *J* = 10.3, 2.4 Hz, 2H), 6.63 (s, 1H), 6.24 (s, 1H), 6.01 (s, 1H), 5.59 (s, 1H), 4.17 – 4.06 (m, 1H), 3.89 (d, *J* = 4.2 Hz, 1H), 3.84 (s, 3H), 3.77 (s, 3H), 3.64 (s, 3H), 3.00 (ddd, *J* = 14.9, 8.8, 5.5 Hz, 1H), 2.70 (dt, *J* = 16.1, 4.4, 4.4 Hz, 1H). **^13^C NMR** (75 MHz, CDCl_3_) δ 147.9, 147.3, 146.7, 145.7, 134.2, 129.2, 126.2, 122.4, 113.9, 111.2, 111.1, 109.8, 79.3, 63.7, 56.0, 55.9, 29.8, 28.4. **HRMS** (APCI^+^, m/z) calculated for C_18_H_21_O_5_ [M+H]^+^:317.13107; found:317.13835.

**1-(3,4-dimethoxyphenyl)-6,7-dimethoxyisochroman (3b)**

The compound was synthesized according to the **General procedure**. Homovanillyl alcohol (59 mg, 0.35 mmol) affords (99 mg, 76% yield). White semi-solid was obtained after column chromatography (SiO_2_, Hexane/EtOAc 80:20). **^1^H NMR** (300 MHz, CDCl_3_) δ 6.83 (s, 3H), 6.65 (s, 1H), 6.25 (s, 1H), 5.62 (s, 1H), 4.14 (ddd, *J* = 11.4, 5.4, 4.1 Hz, 1H), 3.94 – 3.89 (m, 1H), 3.85 (d, *J* = 14.0 Hz, 9H), 3.66 (s, 3H), 3.03 (ddd, *J* = 14.9, 9.0, 5.4 Hz, 1H), 2.72 (dt, *J* = 16.1, 4.2, 4.2 Hz, 1H). **^13^C NMR** (75 MHz, CDCl_3_) δ 149.1, 149.0, 147.95, 147.29, 134.83, 129.17, 126.21, 121.6, 111.7, 111.2, 110.7, 109.8, 79.2, 63.7, 56.0, 56.0, 55.9, 28.4. **HRMS** (APCI^+^, m/z) calculated for C_19_H_23_O_5_ [M+H]^+^: 331.14672; found:331.15415.

**1-(4-fluorophenyl)-6-methoxyisochroman-7-ol (1c)**

The compound was synthesized according to the **General procedure**. Homovanillyl alcohol (0.35 mmol) affords **1c** (80 mg, 84% yield). White semi-solid was obtained after column chromatography (SiO_2_, Hexane/EtOAc 80:20). **^1^H NMR** (300 MHz, CDCl_3_) δ 7.36 – 7.20 (m, 2H), 7.04 (t, *J* = 8.7, 8.7 Hz, 2H), 6.65 (s, 1H), 6.29 (s, 1H), 5.63 (s, 1H), 5.25 (d, *J* = 34.8 Hz, 1H), 4.18 (ddd, *J* = 11.3, 5.5, 3.6 Hz, 1H), 3.97 – 3.90 (m, 1H), 3.89 (s, 3H), 3.08 (ddd, *J* = 15.4, 9.6, 5.5 Hz, 1H), 2.72 (dt, *J* = 16.0, 3.9, 3.9 Hz, 1H). **^13^C NMR** (75 MHz, CDCl_3_) δ 162.6 (d, ^1^J(C-F) = 244.5 Hz), 145.7, 143.8, 138.2 (d, 4J(C-F) = 3.75 Hz), 130.5 (d, ^3^J(C-F) = 8.25 Hz), 129.8, 125.4, 115.3 (d, ^2^J(C-F) = 21.75 Hz), 112.6, 110.6, 78.6, 64.1, 60.5, 56.0, 28.4, 14.3. **HRMS** (APCI^+^, m/z) calculated for C_16_H_16_FO_3_ [M+H]^+^: 275.1005; found: 275.1081.

**1-(4-bromo-3-fluorophenyl)-6-methoxyisochroman-7-ol (1d)**

The compound was synthesized according to the **General procedure**. Homovanillyl alcohol (0.35 mmol) affords **1** (107.2 mg, 87% yield). White semi-solid was obtained after column chromatography (SiO_2_, Hexane/EtOAc 80:20). **^1^H NMR** (300 MHz, CDCl_3_) δ 7.68 (dd, *J* = 6.6, 2.2 Hz, 1H), 7.47 – 7.36 (m, 1H), 7.27 (d, *J* = 8.4 Hz, 1H), 6.82 (s, 1H), 6.45 (s, 1H), 5.76 (s, 1H), 5.69 (s, 1H), 4.32 (ddd, *J* = 11.3, 5.4, 3.7 Hz, 1H), 4.06 (s, 4H), 3.32 – 3.13 (m, 1H), 2.88 (dt, *J* = 16.1, 3.9, 3.9 Hz, 1H).**^13^C NMR** (75 MHz, CDCl_3_) δ 158.8 (d, ^1^J(C-F) = 246 Hz), 145.8, 143.9, 139.9 (d, ^4^J(C-F) = 3.75 Hz), 133.8, 129.4 (d, ^3^J(C-F) = 7.5 Hz), 129.0, 125.4, 116.3 (d, ^2^J(C-F) = 21.75 Hz), 112.4, 110.7, 109.1 (d, ^2^J(C-F) = 21.75 Hz), 78.1, 64., 56.06, 28.3. **HRMS** (APCI^+^, m/z) calculated for C_16_H_15_BrFO_3_ [M+H]^+^: 353.0110; found: 353.0042.

**6-methoxy-1-(4-nitrophenyl)isochroman-7-ol (1e)**

The compound was synthesized according to the **General procedure**. Homovanillyl alcohol (0.35 mmol) affords **1e** (66.4 mg, 63% yield). White semi-solid was obtained after column chromatography (SiO_2_, Hexane/EtOAc 80:20). **^1^H NMR** (300 MHz, CDCl_3_) δ 8.19 (d, *J* = 8.7 Hz, 2H), 7.49 (d, *J* = 8.7 Hz, 2H), 6.65 (s, 1H), 6.22 (s, 1H), 5.71 (s, 1H), 5.49 (s, 1H), 4.16 (ddd, *J* = 11.3, 5.5, 3.5 Hz, 1H), 3.92 (dd, *J* = 9.8, 4.0 Hz, 1H), 3.88 (s, 3H), 3.08 (ddd, *J* = 15.5, 9.7, 5.5 Hz, 1H), 2.72 (dt, *J* = 16.1, 3.8, 3.8 Hz, 1H). **^13^C NMR** (75 MHz, CDCl_3_) δ 149.5, 147.7, 145.9, 144.1, 129.6, 128.5, 125.3, 123.7, 112.2, 110.8, 78.3, 64.3, 56.0, 28.3. **HRMS** (APCI^+^, m/z) calculated for C_16_H_16_NO_5_ [M+H]^+^: 302.09502; found: 302.1023.

**1-(2-bromophenyl)-6-methoxyisochroman-7-ol (1f)**

The compound was synthesized according to the **General procedure**. Homovanillyl alcohol (59 mg, 0.35 mmol) affords **1f** (97 mg, 83% yield). White semi-solid was obtained after column chromatography (SiO_2_, Hexane/EtOAc 80:20). **^1^H NMR** (300 MHz, MeOD) δ 7.61 (dd, *J* = 7.8, 1.4 Hz, 1H), 7.30 – 7.10 (m, 3H), 6.71 (s, 1H), 6.10 (d, *J* = 15.9 Hz, 2H), 4.11 (ddd, *J* = 11.3, 5.5, 3.6 Hz, 1H), 3.91 – 3.83 (m, 1H), 3.82 (s, 3H), 3.02 (ddd, *J* = 15.6, 9.7, 5.6 Hz, 1H), 2.68 (dt, *J* = 16.1, 3.9, 3.9 Hz, 1H). **^13^C NMR** (75 MHz, CD_3_OD) δ 147.3, 145.0, 142.1, 132.9, 131.3, 129.8, 129.0, 127.8, 125.3, 124.4, 112.9, 111.7, 78.1, 64.4, 55.4, 28.3. **HRMS** (APCI^+^, m/z) calculated for C_16_H_16_BrO_3_ [M+H]^+^: 335.02046; found:335.0268.

**1-(4-bromophenyl)-6-methoxyisochroman-7-ol (1g)**

The compound was synthesized according to the **General procedure**. Homovanillyl alcohol (0.35 mmol) affords **1g** (86 mg, 74% yield). White semi-solid was obtained after column chromatography (SiO_2_, Hexane/EtOAc 80:20). **^1^H NMR** (300 MHz, CDCl_3_) δ 7.52 (d, *J* = 8.4 Hz, 2H), 7.24 (d, *J* = 8.4 Hz, 2H), 6.68 (s, 1H), 6.32 (s, 1H), 5.64 (s, 1H), 5.55 (s, 1H), 4.20 (ddd, *J* = 10.9, 5.3, 3.6 Hz, 1H), 3.93 (s, 4H), 3.10 (ddd, *J* = 15.5, 9.5, 5.5 Hz, 1H), 2.76 (dt, *J* = 16.1, 4.0, 4.0 Hz, 1H). **^13^C NMR** (75 MHz, CDCl_3_) δ 145.7, 143.9, 141.3, 131.6, 130.5, 129.4, 125.3, 122.1, 112.5, 110.6, 78.6, 64.1, 56.0, 28.4. **HRMS** (APCI^+^, m/z) calculated for C_16_H_16_BrO_3_ [M+H]^+^: 335.02046; found:335.0276.

**6-methoxy-1-(4-methoxyphenyl) isochroman-7-ol (1h)**

The compound was synthesized according to the **General procedure**. Homovanillyl alcohol (0.35 mmol) affords **1h** (60.1 mg, 60% yield). White semi-solid was obtained after column chromatography (SiO_2_, Hexane/EtOAc 80:20). **^1^H NMR** (300 MHz, CDCl_3_) δ 7.22 (d, *J* = 8.5 Hz, 2H), 6.86 (d, *J* = 8.6 Hz, 2H), 6.62 (s, 1H), 6.31 (s, 1H), 5.59 (s, 1H), 5.47 (s, 1H), 4.22 – 4.08 (m, 1H), 3.87 (s, 4H), 3.80 (s, 3H), 3.04 (ddd, *J* = 15.2, 9.4, 5.7 Hz, 1H), 2.69 (dt, *J* = 16.0, 4.0, 4.0 Hz, 1H). **^13^C NMR** (75 MHz, CDCl_3_) δ 159.5, 145.5, 143.8, 134.6, 130.4, 130.1, 125.4, 113.8, 112.8, 110.5, 78.9, 63.9, 56.0, 55.4, 28.5 **HRMS** (APCI^+^, m/z) calculated for C_17_H_19_O_4_ [M+H]^+^: 287.12051; found: 287.1284.

**1-(3,4-dihydroxyphenyl)isochroman-6,7-diol (2i, Blapsin B)**

The compound was synthesized according to the **General procedure**. 3-hydroxytyrosol (0.35 mmol) affords **2i** (**blapsin B)** (57.5 mg, 60% yield). White semi-solid was obtained after column chromatography (SiO_2_, Hexane/EtOAc 80:20). **^1^H NMR** (300 MHz, MeOD) δ 6.74 (d, *J* = 8.0 Hz, 1H), 6.68 – 6.61 (m, 2H), 6.55 (s, 1H), 6.16 (s, 1H), 5.45 (s, 1H), 4.13 – 3.99 (m, 1H), 3.86 – 3.73 (m, 1H), 2.90 (ddd, *J* = 14.7, 9.3, 5.5 Hz, 1H), 2.60 (dt, *J* = 16.0, 4.2, 4.2 Hz, 1H).**^13^C NMR** (75 MHz, CD_3_OD) δ 145.4, 145.2, 144.3, 143.6, 134.5, 129.0, 125.2, 121.0, 116.2, 115.4, 114.9, 113.7, 79.7, 63.9, 28.2. **HRMS** (APCI^+^, m/z) calculated for C_15_H_15_O_5_ [M+H]^+^: 275.0841; found: 275.0914

**6-methoxyisochroman-7-ol (1j)**

The compound was synthesized according to the **General procedure**. Homovanillyl alcohol (0.35 mmol) affords **1j** (37.8 mg, 60% yield). White semi-solid was obtained after column chromatography (SiO_2_, Hexane/EtOAc 80:20). **^1^H NMR** (300 MHz, CDCl_3_) δ 6.59 (s, 1H), 6.54 (s, 1H), 5.55 (s, 1H), 4.67 (s, 2H), 3.94 (t, *J* = 5.7, 5.7 Hz, 2H), 3.86 (s, 3H), 2.76 (t, *J* = 5.1, 5.1 Hz, 2H).**^13^C NMR** (75 MHz, CDCl_3_) δ 145.4, 144.1, 127.5, 124.5, 111.0, 110.2, 67.7, 65.5, 56.1, 29.8. **HRMS** (APCI^+^, m/z) calculated for C_10_H_13_O_3_ [M+H]^+^: 181.0786; found: 181.0855 .

**1-ethyl-6-methoxyisochroman-7-ol (1k)**

The compound was synthesized according to the **General procedure**. Homovanillyl alcohol (0.35 mmol) affords **1k** (59.7 mg, 82% yield). White semi-solid was obtained after column chromatography (SiO_2_, Hexane/EtOAc 80:20). **^1^H NMR** (300 MHz, CDCl_3_) δ 6.64 (s, 1H), 6.57 (s, 1H), 5.53 (s, 1H), 4.66 – 4.55 (m, 1H), 4.11 (ddd, *J* = 11.2, 5.3, 3.4 Hz, 1H), 3.86 (s, 3H), 3.73 (ddd, *J* = 11.2, 9.8, 3.7 Hz, 1H), 2.99 – 2.80 (m, 1H), 2.57 (dt, *J* = 15.9, 3.6, 3.6 Hz, 1H), 1.91 (dtd, *J* = 14.7, 7.4, 7.3, 3.3 Hz, 1H), 1.75 (dq, *J* = 21.5, 7.4, 7.4, 7.4 Hz, 1H), 0.96 (t, *J* = 7.3, 7.3 Hz, 3H). **^13^C NMR** (75 MHz, CDCl_3_) δ 145.1, 144.0, 131.0, 125.6, 110.8, 76.6, 63.5, 56.0, 28.9, 28.7, 9.5. **HRMS** (APCI^+^, m/z) calculated for C_12_H_17_O_3_ [M+H]^+^: 209.1099; found: 209.1173.

**6-methoxy-1-propylisochroman-7-ol (1l)**

The compound was synthesized according to the **General procedure**. Homovanillyl alcohol (0.35 mmol) affords **1l** (66.1 mg, 85% yield). White semi-solid was obtained after column chromatography (SiO_2_, Hexane/EtOAc 80:20). **^1^H NMR** (300 MHz, CDCl_3_) δ 6.64 (s, 1H), 6.57 (s, 1H), 5.53 (s, 1H), 4.66 (dd, *J* = 7.9, 3.6 Hz, 1H), 4.11 (ddd, *J* = 11.2, 5.3, 3.6 Hz, 1H), 3.86 (s, 3H), 3.73 (ddd, *J* = 11.2, 9.6, 3.8 Hz, 1H), 2.98 – 2.82 (m, 1H), 2.58 (dt, *J* = 15.9, 3.7, 3.7 Hz, 1H), 1.84 – 1.69 (m, 2H), 1.54 – 1.39 (m, 2H), 0.94 (t, *J* = 7.4, 7.4 Hz, 3H). **^13^C NMR** (75 MHz, CDCl_3_) δ 144.9, 143.8, 131.2, 125.3, 110.7, 110.7, 74.4, 63.3, 55.9, 38.1, 28.7, 18.3, 13.6. **HRMS** (APCI^+^, m/z) calculated for C_13_H_19_O_3_ [M+H]^+^: 223.1255; found: 223.1325.

**1-(sec-butyl)-6-methoxyisochroman-7-ol (1m)**

The compound was synthesized according to the **General procedure**. Homovanillyl alcohol (0.35 mmol) affords **1m** (57 mg, 69% yield). White semi-solid was obtained after column chromatography (SiO_2_, Hexane/EtOAc 80:20). **^1^H NMR** (300 MHz, CDCl_3_) δ 6.65 (d, *J* = 2.2 Hz, 1H), 6.56 (s, 1H), 5.50 (d, *J* = 2.7 Hz, 1H), 4.63 (d, *J* = 22.6 Hz, 1H), 4.14 (ddd, *J* = 11.0, 5.6, 1.6 Hz, 1H), 3.87 (s, 3H), 3.70 – 3.56 (m, 1H), 3.04 – 2.86 (m, 1H), 2.45 (d, *J* = 15.8 Hz, 1H), 1.93 – 1.80 (m, 1H), 1.66 – 1.57 (m, 1H), 1.45 (dt, *J* = 13.4, 7.7, 7.7 Hz, 1H), 1.11 (d, *J* = 6.9 Hz, 2H), 1.00 (t, *J* = 7.4, 7.4 Hz, 2H), 0.68 (d, *J* = 6.8 Hz, 2H). **^13^C NMR** (75 MHz, CDCl_3_) δ 145.7, 143.2, 130.7, 126.5, 110.6, 110.4, 78.17, 64.4, 55.8, 40.2, 29.7, 26.7, 16.4, 12.7. **HRMS** (APCI^+^, m/z) calculated for C_14_H_21_O_3_ [M+H]^+^: 237.1412; found:237.1483 .

**1-butyl-6-methoxyisochroman-7-ol (1n)**

The compound was synthesized according to the **General procedure**. Homovanillyl alcohol (0.35 mmol) affords **1n** (63.6 mg, 77% yield). White semi-solid was obtained after column chromatography (SiO_2_, Hexane/EtOAc 80:20). **^1^H NMR** (300 MHz, CDCl_3_) δ 6.65 (s, 1H), 6.57 (s, 1H), 5.53 (s, 1H), 4.68 – 4.60 (m, 1H), 4.11 (ddd, *J* = 11.2, 5.3, 3.5 Hz, 1H), 3.86 (s, 3H), 3.73 (ddd, *J* = 11.2, 9.7, 3.8 Hz, 1H), 2.90 (ddd, *J* = 14.9, 9.7, 5.3 Hz, 1H), 2.58 (dt, *J* = 15.9, 3.7, 3.7 Hz, 1H), 1.89 – 1.66 (m, 2H), 1.49 – 1.31 (m, 4H), 0.91 (t, *J* = 7.1, 7.1 Hz, 3H). **^13^C NMR** (75 MHz, CDCl_3_) δ 145.1, 144.0, 131.3, 125.4, 110.8, 110.8, 75.6, 62.9, 56.0, 35.7, 28.9, 27.4, 22.9, 14.2. **HRMS** (APCI^+^, m/z) calculated for C_14_H_21_O_3_ [M+H]^+^: 237.1412; found: 237.1485.

.

**10. Spectral data of isolated compounds**

**Figure S3**: ^1^H and ^13^C NMR spectra (CDCl_3_) of compound **1a**.

**Figure S4**: ^1^H and ^13^C NMR spectra (CDCl_3_) of compound **1b**.

**Figure S5**: ^1^H and ^13^C NMR spectra (CDCl_3_) of compound **3a**.

**Figure S6**: ^1^H and ^13^C NMR spectra (CDCl_3_) of compound **3b**.

**Figure S7**: ^1^H and ^13^C NMR spectra (CDCl_3_) of compound **1c**.

**Figure S8**: ^1^H and ^13^C NMR spectra (CDCl_3_) of compound **1d**.

**Figure S9**: ^1^H and ^13^C NMR spectra (CDCl_3_) of compound **1e**.

**Figure S10**: ^1^H and ^13^C NMR spectra (CD_3_OD) of compound **1f**.

**Figure S11**: ^1^H and ^13^C NMR spectra (CDCl_3_) of compound **1g**.

**Figure S12**: ^1^H and ^13^C NMR spectra (CDCl_3_) of compound **1h**.

**Figure S13**: ^1^H and ^13^C NMR spectra (CD_3_OD) of compound **2i** **blapsin B**.

**Figure S14:** ^1^H and ^13^C NMR spectra (CDCl_3_) of compound **1j**.

**Figure S15**: ^1^H and ^13^C NMR spectra (CDCl_3_) of compound **1k**.

**Figure S16**: ^1^H and ^13^C NMR spectra (CDCl_3_) of compound **1l**.

**Figure S17**: ^1^H and ^13^C NMR spectra (CDCl_3_) of compound **1m**.

**Figure S18**: ^1^H and ^13^C NMR spectra (CDCl_3_) of compound **1n**.

**11. References**

[1]	a) H. Qin, X. Hu, J. Wang, H. Cheng, L. Chen, Z. Qi, *Green Energy Environm.* **2020**, *5*, 8; b) Q. Zhang, K. de Oliveira Vigier, S. Royer, F. Jérôme, *Chem. Soc. Rev.* **2012**, *41*, 7108.

[2]	A. de Santi, M. V. Galkin, C. W. Lahive, P. J. Deuss, K. Barta, *ChemSusChem* **2020**, *13*, 4468.

[3]	A. M. Afanasenko, X. Wu, A. de Santi, W. A. M. Elgaher, A. M. Kany, R. Shafiei, M.-S. Schulze, T. F. Schulz, J. Haupenthal, A. K. H. Hirsch and K. Barta , *Angew Chem Int Ed* **2023**.

[4]	M. M. Boucher, M. H. Furigay, P. K. Quach, C. S. Brindle, *Org. Process Res. Dev.* **2017**, *21*, 1394.

[5]	G. Spano, S. Ramello, G. Girotti, F. Rivetti, A. Carati, WO2006002805A1, **2006**.

[6]	H. Bartkowiak, B. Haase, R. Hofmann, H. J. Naumann, B. Raue, DD258531A3., **1988**.

[7]	M. S. Kharasch, A. Fono, W. Nudenberg, *J. Org. Chem.* **1950**, *15*, 748.

[8]	S. Umemura, N. Takamitsu, T. Hamamoto, N. Kuroda, US4078006A, **1978**.

[9]	T. G. Gant, S. Sarshar, AU2008266124, **2008**.

[10]	S. Swaninathan, G. B. V. Ramanarayanan, WO2015/15445, **2015**.

[11]	Z. Yang, F. Tan, H. Wong, P. Hanselmann, WO2012/3625, **2012**.

[12]	H.R. Bjørsvik, L. Liguori, F. Minisci, *Org. Process Res. Dev.* **2000**, *4*, 534.

[13]	R. Bernini, F. Crisante, F. D’Acunzo, P. Gentili, E. Ussia, *New J. Chem.* **2016**, *40*, 3314.

[14]	V. E. Tarabanko and D. V. Pethukov **2003**.

[15]	J. Zhou, C. Wang, D. Xue, W. Tang, J. Xiao, C. Li, *Tetrahedron* **2018**, *74*, 7040.

[16]	N. Kapadia, W. Harding, *Tetrahedron* **2013**, *69*.

[17]	C. Muller, F. Horký, M. Vayer, A. Golushko, D. Lebœuf, J. Moran, *Chem. Sci.* **2023**, *14*, 2983.

[18]	M. Guiso, A. Betrow, C. Marra, *Eur J Org Chem* **2008**, *2008*, 1967.

[19]	M. Guiso, A. Bianco, C. Marra, C. Cavarischia, *Eur J Org Chem* **2003**, *2003*, 3407.

[20]	P. Lorenz, M. Zeh, J. Martens-Lobenhoffer, H. Schmidt, G. Wolf, T. F. W. Horn, *Free Radic. Res.* **2005**, *39*, 535.

[21]	B. Bouguerne, P. Hoffmann, C. Lherbet, *Synth. Commun.* **2010**, *40*, 915.

[22]	N. Tanaka, S. R. Mitton-Fry, M. U. Hwang, J. L. Zhu, K. A. Scheidt, *ACS Catal.* **2024**, *14*, 7949.

[23]	A. Saeed, *CCL* **2010**, *21*, 261.

[24]	S. Sarkar, S. Sultana, K. Indukuri, R. Unnava, A. Saikia, *Synthesis* **2016**, *48*, 1727.

[25]	W. H. Miles, S. K. Heinsohn, M. K. Brennan, D. T. Swarr, P. M. Eidam, K. A. Gelato, *Synthesis* **2002**, 1541.

[26]	A. K. Verma, A. Chennaiah, S. Dubbu, Y. D. Vankar, *Org.Biomol. Chem.* **2018**, *16*, 8258.

[27]	B. Bouguerne, C. Lherbet, M. Baltas, *LOC* **2010**, *7*, 420.

[28]	A. Saito, M. Takayama, A. Yamazaki, J. Numaguchi, Y. Hanzawa, *Tetrahedron* **2007**, *63*, 4039.

[29]	C. R. Reddy, A. G. Burra, K. K. Singarapu, R. Grée, *Eur J Org Chem* **2016**, *2016*, 5274.

[30]	Z. Khorsandi, A. R. Khosropour, V. Mirkhani, I. Mohammadpoor-Baltork, M. Moghadam, S. Tangestaninejad, *Tetrahedron Letters* **2011**, *52*, 1213.
